# Supplementary material for: The Life Expectancy Gap between Registered Disabled and Non-Disabled People in Korea from 2004 to 2017
Source: Int J Environ Res Public Health. 2019 Jul 20;16(14):2593. doi: 10.3390/ijerph16142593 (PMC6678634; doi:10.3390/ijerph16142593)
Supplement: Supplementary file 1 [file ijerph-16-02593-s001.pdf]

Supplementary Table 1. Numbers of population and deaths in registered disabled and non-disabled people in Korea between 2004 and 2017.

Year	Overall		Non-disabled		Registered disabled people		% of registered disabled population
	No. of population	No. of deaths	No. of population	No. of deaths	No. of population	No. of deaths	
Men and women combined							
2004	48,329,061	237,419	46,687,075	199,303	1,641,986	38,116	3.4%
2005	48,530,981	237,912	46,708,359	195,395	1,822,622	42,517	3.8%
2006	48,737,480	236,995	46,727,959	189,435	2,009,521	47,560	4.1%
2007	48,967,709	240,047	46,793,167	188,083	2,174,542	51,964	4.4%
2008	49,245,374	239,798	46,909,805	183,719	2,335,569	56,079	4.7%
2009	49,519,213	240,935	47,016,394	181,620	2,502,819	59,315	5.1%
2010	49,756,545	249,930	47,174,120	186,020	2,582,425	63,910	5.2%
2011	49,980,840	252,191	47,379,616	187,726	2,601,224	64,465	5.2%
2012	50,228,255	262,277	47,631,381	195,087	2,596,874	67,190	5.2%
2013	50,455,808	259,431	47,872,110	193,709	2,583,698	65,722	5.1%
2014	50,658,369	262,816	48,084,642	197,048	2,573,727	65,768	5.1%
2015	50,855,927	269,976	48,280,482	201,701	2,575,445	68,275	5.1%
2016	51,042,438	271,023	48,446,326	202,602	2,596,112	68,421	5.1%
2017	51,195,634	276,028	48,570,282	205,866	2,625,352	70,162	5.1%
Sum	697,503,634	3,536,778	664,281,718	2,707,314	33,221,916	829,464	4.8%
Men							
2004	24,243,142	130,834	23,171,643	105,293	1,071,499	25,541	4.4%
2005	24,338,398	130,372	23,173,846	102,454	1,164,552	27,918	4.8%
2006	24,438,669	130,038	23,182,482	99,439	1,256,187	30,599	5.1%
2007	24,549,015	131,617	23,216,531	98,982	1,332,484	32,635	5.4%
2008	24,681,837	132,347	23,277,313	97,260	1,404,524	35,087	5.7%
2009	24,814,033	133,287	23,337,261	96,585	1,476,772	36,702	6.0%
2010	24,922,943	138,129	23,411,987	99,397	1,510,956	38,732	6.1%
2011	25,023,245	139,216	23,504,794	100,165	1,518,451	39,051	6.1%
2012	25,136,066	143,509	23,622,062	103,305	1,514,004	40,204	6.0%
2013	25,237,439	141,885	23,732,718	102,790	1,504,721	39,095	6.0%
2014	25,327,681	143,549	23,830,143	104,709	1,497,538	38,840	5.9%
2015	25,415,837	146,065	23,917,298	106,117	1,498,539	39,948	5.9%
2016	25,498,033	146,293	23,989,349	106,411	1,508,684	39,882	5.9%
2017	25,563,384	148,139	24,040,239	107,480	1,523,145	40,659	6.0%
Sum	349,189,722	1,935,280	329,407,666	1,430,387	19,782,056	504,893	5.7%
Women							

2004	24,085,919	106,585	23,515,432	94,010	570,487	12,575	2.4%
2005	24,192,583	107,540	23,534,513	92,941	658,070	14,599	2.7%
2006	24,298,811	106,957	23,545,477	89,996	753,334	16,961	3.1%
2007	24,418,694	108,430	23,576,636	89,101	842,058	19,329	3.4%
2008	24,563,537	107,451	23,632,492	86,459	931,045	20,992	3.8%
2009	24,705,180	107,648	23,679,133	85,035	1,026,047	22,613	4.2%
2010	24,833,602	111,801	23,762,133	86,623	1,071,469	25,178	4.3%
2011	24,957,595	112,975	23,874,822	87,561	1,082,773	25,414	4.3%
2012	25,092,189	118,768	24,009,319	91,782	1,082,870	26,986	4.3%
2013	25,218,369	117,546	24,139,392	90,919	1,078,977	26,627	4.3%
2014	25,330,688	119,267	24,254,499	92,339	1,076,189	26,928	4.2%
2015	25,440,090	123,911	24,363,184	95,584	1,076,906	28,327	4.2%
2016	25,544,405	124,730	24,456,977	96,191	1,087,428	28,539	4.3%
2017	25,632,250	127,889	24,530,043	98,386	1,102,207	29,503	4.3%
Sum	348,313,912	1,601,498	334,874,052	1,276,927	13,439,860	324,571	3.9%

Supplementary Table 2. Numbers of population and deaths among registered disabled people in Korea between 2004 and 2017 according to disability grade (grade 1-6)

Year	Grade 1		Grade 2		Grade 3		Grade 4		Grade 5		Grade 6	
	No. of population	No. of deaths	No. of population	No. of deaths	No. of population	No. of deaths	No. of population	No. of deaths	No. of population	No. of deaths	No. of population	No. of deaths
Men and women combined												
2004	183,445	9,536	293,301	10,065	287,840	6,510	230,539	4,132	289,222	4,274	357,639	3,599
2005	209,688	10,662	310,970	10,802	307,410	7,293	253,019	4,665	332,293	5,049	409,242	4,046
2006	226,599	12,064	332,591	11,684	331,533	7,913	278,426	5,242	380,369	5,979	460,003	4,678
2007	242,679	13,106	349,287	12,547	353,998	8,499	303,729	5,766	424,647	6,827	500,202	5,219
2008	257,038	13,875	359,724	13,047	377,653	9,205	330,757	6,313	471,118	7,870	539,279	5,769
2009	269,805	14,004	367,131	13,169	405,556	9,878	362,590	7,159	521,138	8,795	576,599	6,310
2010	274,380	14,622	363,908	13,636	419,665	10,771	383,475	7,992	537,255	9,923	603,742	6,966
2011	270,446	13,501	355,362	13,471	423,772	10,901	392,111	8,842	541,613	10,231	617,920	7,519
2012	264,153	12,965	348,566	13,847	421,772	11,401	391,259	9,787	542,432	11,010	628,692	8,180
2013	258,416	12,013	342,239	13,264	419,516	10,898	386,495	9,824	540,393	11,116	636,639	8,607
2014	254,316	11,181	338,102	13,200	418,167	10,931	381,853	10,097	538,202	11,363	643,087	8,996
2015	252,130	11,518	337,104	13,290	416,294	11,213	376,501	10,591	541,736	12,038	651,680	9,625
2016	250,267	11,489	337,858	13,083	417,768	11,012	379,427	10,715	551,248	12,145	659,544	9,977
2017	248,706	11,285	339,351	13,251	419,917	11,022	385,167	11,378	566,066	12,985	666,145	10,241
Sum	3,462,068	171,821	4,775,494	178,356	5,420,861	137,447	4,835,348	112,503	6,777,732	129,605	7,950,413	99,732
Men												
2004	110,542	5,826	177,415	6,322	193,256	4,710	149,962	2,970	184,255	2,933	256,069	2,780
2005	125,557	6,274	186,493	6,744	203,848	5,295	158,459	3,236	203,129	3,324	287,066	3,045
2006	134,617	6,997	197,535	7,185	216,640	5,557	167,255	3,583	223,365	3,855	316,775	3,422
2007	143,727	7,507	206,140	7,614	228,118	5,877	174,285	3,747	240,589	4,118	339,625	3,772
2008	151,824	7,975	211,172	7,950	240,231	6,345	181,459	4,057	258,328	4,714	361,510	4,046
2009	159,074	8,137	214,561	8,015	254,315	6,641	189,514	4,418	276,863	5,068	382,445	4,423
2010	161,483	8,323	211,969	8,160	260,331	7,203	196,244	4,765	284,698	5,543	396,231	4,738
2011	159,498	7,620	206,629	8,042	260,613	7,181	200,847	5,191	287,902	5,841	402,962	5,176
2012	156,031	7,264	202,673	8,290	258,559	7,455	200,407	5,659	288,703	6,150	407,631	5,386
2013	152,918	6,827	199,097	7,836	256,205	7,017	197,745	5,548	287,780	6,166	410,976	5,701
2014	150,453	6,230	197,100	7,734	254,613	6,985	195,320	5,736	286,798	6,258	413,254	5,897
2015	149,154	6,474	196,966	7,862	253,097	7,099	193,679	5,869	288,509	6,494	417,134	6,150
2016	147,945	6,366	197,544	7,794	253,471	6,898	195,627	5,789	293,771	6,628	420,326	6,407

2017	146,993	6,356	198,677	7,762	254,225	6,952	199,401	6,085	301,224	7,012	422,625	6,492
Sum	2,049,816	98,176	2,803,971	107,310	3,387,522	91,215	2,600,204	66,653	3,705,914	74,104	5,234,629	67,435
Women												
2004	72,903	3,710	115,886	3,743	94,584	1,800	80,577	1,162	104,967	1,341	101,570	819
2005	84,131	4,388	124,477	4,058	103,562	1,998	94,560	1,429	129,164	1,725	122,176	1,001
2006	91,982	5,067	135,056	4,499	114,893	2,356	111,171	1,659	157,004	2,124	143,228	1,256
2007	98,952	5,599	143,147	4,933	125,880	2,622	129,444	2,019	184,058	2,709	160,577	1,447
2008	105,214	5,900	148,552	5,097	137,422	2,860	149,298	2,256	212,790	3,156	177,769	1,723
2009	110,731	5,867	152,570	5,154	151,241	3,237	173,076	2,741	244,275	3,727	194,154	1,887
2010	112,897	6,299	151,939	5,476	159,334	3,568	187,231	3,227	252,557	4,380	207,511	2,228
2011	110,948	5,881	148,733	5,429	163,159	3,720	191,264	3,651	253,711	4,390	214,958	2,343
2012	108,122	5,701	145,893	5,557	163,213	3,946	190,852	4,128	253,729	4,860	221,061	2,794
2013	105,498	5,186	143,142	5,428	163,311	3,881	188,750	4,276	252,613	4,950	225,663	2,906
2014	103,863	4,951	141,002	5,466	163,554	3,946	186,533	4,361	251,404	5,105	229,833	3,099
2015	102,976	5,044	140,138	5,428	163,197	4,114	182,822	4,722	253,227	5,544	234,546	3,475
2016	102,322	5,123	140,314	5,289	164,297	4,114	183,800	4,926	257,477	5,517	239,218	3,570
2017	101,713	4,929	140,674	5,489	165,692	4,070	185,766	5,293	264,842	5,973	243,520	3,749
Sum	1,412,252	73,645	1,971,523	71,046	2,033,339	46,232	2,235,144	45,850	3,071,818	55,501	2,715,784	32,297

Supplementary Table 3. Time trends of life expectancy differences between non-disabled and registered disabled people and gender differences from 2004 to 2017

Year	Difference between non-disabled and registered disabled people			Gender difference (women-men)		
	Men and women combined	Men	Women	Non-disabled	Disabled	Difference between non-disabled and registered disabled people
2004	20.4	18.6	20.3	6.4	4.7	1.7
2005	20.9	18.8	21.8	6.0	3.0	3.0
2006	20.5	18.9	20.3	5.9	4.5	1.4
2007	19.7	17.3	20.7	6.0	2.6	3.4
2008	19.0	17.5	19.1	5.9	4.3	1.6
2009	18.3	16.3	19.0	5.9	3.2	2.7
2010	18.3	16.5	19.0	6.0	3.5	2.5
2011	17.8	15.3	19.8	6.0	1.5	4.5
2012	17.2	15.9	17.1	5.9	4.7	1.2
2013	16.2	15.1	15.7	5.8	5.2	0.6
2014	16.7	15.5	16.8	5.7	4.4	1.3
2015	17.1	15.9	17.2	5.6	4.3	1.3
2016	15.9	13.8	17.2	5.6	2.2	3.4
2017	16.4	15.0	16.6	5.5	3.9	1.6
Trends	<.0001	<.0001	<.0001	<.0001	0.9678	0.5701

For trend analyses of data during the entire time period are assessed by the least squares regression method.

Supplementary Table 4. Life expectancy of people with registered disabilities according to disability grades (grade 1-6) in Korea between 2004 and 2017

Year	Grade 1	Grade 2	Grade 3	Grade 4	Grade 5	Grade 6
**Men and women**						
2004	40.7	57.4	65.3	65.9	71.1	75.1
2005	40.8	58.1	64.6	71.5	71.3	76.5
2006	43.0	56.7	64.8	70.5	70.4	75.7
2007	43.8	58.9	64.9	72.6	72.3	78.0
2008	44.5	61.0	65.0	73.6	73.2	78.4
2009	46.6	60.5	65.4	72.3	74.4	78.8
2010	46.3	62.0	67.2	73.0	72.8	78.0
2011	48.1	63.7	68.3	74.1	67.6	79.2
2012	48.6	63.1	68.4	70.1	74.3	76.7
2013	50.4	63.6	69.9	73.2	74.9	79.0
2014	50.2	62.8	69.5	73.3	75.5	77.7
2015	49.3	63.1	70.6	70.1	75.1	78.8
2016	50.8	64.6	70.4	72.2	77.5	79.6
2017	49.7	64.4	69.8	71.7	78.1	77.7
**Men**						
2004	38.8	55.9	63.6	65.7	71.2	75.1
2005	40.0	57.1	62.2	69.4	70.6	74.0
2006	42.2	54.1	63.4	68.1	66.5	73.8
2007	43.5	58.5	62.5	68.8	69.3	76.1
2008	43.6	59.8	63.2	69.2	68.8	76.8
2009	46.5	57.8	64.8	70.4	70.3	75.4
2010	46.1	60.9	65.1	69.2	72.4	74.4
2011	48.7	62.6	67.2	70.9	64.1	76.1
2012	47.9	62.0	65.5	64.2	70.2	74.5
2013	49.4	62.0	67.3	69.5	72.9	76.7
2014	49.0	61.8	69.2	69.1	72.3	76.3
2015	48.7	61.5	68.6	65.8	71.9	77.5
2016	51.0	63.5	69.6	69.6	75.5	77.3
2017	48.5	63.4	68.7	68.3	75.5	76.5
**Women**						
2004	44.1	60.1	69.4	66.5	71.5	76.4
2005	42.3	59.7	69.8	74.9	71.7	81.5
2006	44.7	61.1	68.3	74.3	75.9	79.0
2007	44.7	59.6	69.6	78.0	76.4	81.2

2008	46.0	63.0	68.6	80.4	78.3	81.3
2009	47.3	64.9	67.2	72.7	78.6	84.4
2010	47.1	64.3	70.8	77.4	73.6	83.5
2011	47.5	65.3	70.1	77.3	71.3	83.7
2012	50.0	64.8	73.2	76.7	79.6	80.1
2013	52.4	66.4	74.2	77.4	76.6	82.7
2014	52.3	64.4	71.0	78.2	79.6	79.5
2015	50.2	65.7	74.2	75.8	78.5	80.6
2016	50.4	66.2	71.8	74.7	79.8	83.2
2017	51.8	66.4	72.3	75.1	80.7	79.7

Supplementary Table 5. Life expectancy differences (years) between non-disabled and registered disabled people in Korea according to their disability grade between 2004 and 2017

Year	Grade 1	Grade 2	Grade 3	Grade 4	Grade 5	Grade 6
**Men and women**						
2004	38.6	21.9	14.0	13.4	8.2	4.2
2005	38.9	21.7	15.2	8.3	8.4	3.3
2006	37.3	23.6	15.5	9.8	9.9	4.6
2007	36.8	21.8	15.8	8.1	8.4	2.7
2008	36.8	20.3	16.4	7.7	8.1	2.9
2009	35.2	21.3	16.4	9.4	7.3	3.0
2010	35.7	20.0	14.8	9.0	9.2	4.1
2011	34.3	18.7	14.1	8.3	14.9	3.2
2012	33.9	19.5	14.1	12.5	8.2	5.9
2013	32.7	19.5	13.3	10.0	8.2	4.2
2014	33.2	20.6	13.9	10.1	7.9	5.7
2015	34.5	20.7	13.2	13.7	8.7	5.0
2016	33.4	19.6	13.8	11.9	6.7	4.6
2017	34.6	20.0	14.6	12.7	6.3	6.7
**Men**						
2004	37.0	19.9	12.3	10.2	4.6	0.7
2005	36.5	19.4	14.2	7.1	5.9	2.5
2006	34.9	22.9	13.6	8.9	10.5	3.2
2007	33.8	18.8	14.9	8.5	8.0	1.2
2008	34.3	18.2	14.8	8.8	9.2	1.2
2009	31.9	20.6	13.7	8.0	8.1	3.1
2010	32.6	17.8	13.6	9.5	6.3	4.2
2011	30.3	16.4	11.8	8.1	14.9	2.9
2012	31.3	17.3	13.7	15.0	9.0	4.7
2013	30.4	17.9	12.5	10.3	6.9	3.1
2014	31.1	18.4	11.0	11.0	7.9	3.9
2015	31.9	19.2	12.1	14.9	8.7	3.1
2016	30.0	17.5	11.4	11.4	5.5	3.7
2017	32.8	17.9	12.6	13.0	5.8	4.8
**Women**						
2004	38.1	22.1	12.7	15.7	10.6	5.8
2005	40.2	22.7	12.7	7.5	10.8	1.0
2006	38.3	21.8	14.6	8.6	7.0	3.9
2007	38.6	23.7	13.8	5.3	6.9	2.1

2008	37.9	20.9	15.3	3.5	5.7	2.6
2009	37.0	19.5	17.1	11.6	5.7	-0.1
2010	37.6	20.3	13.8	7.3	11.0	1.1
2011	37.5	19.7	14.9	7.7	13.7	1.2
2012	35.1	20.3	11.9	8.4	5.5	5.1
2013	33.2	19.2	11.4	8.2	9.0	2.9
2014	33.6	21.4	14.9	7.7	6.3	6.4
2015	36.0	20.5	12.0	10.4	7.7	5.6
2016	36.2	20.4	14.8	11.9	6.8	3.4
2017	35.0	20.4	14.6	11.8	6.2	7.1
